# Dexamethasone 8 mg for Cancer-Related Fatigue in Inpatients with Advanced Cancer Undergoing Palliative Care: A Multicenter Phase II Trial

**DOI:** 10.1089/pmr.2021.0053

**Published:** 2021-11-17

**Authors:** Tomofumi Miura, Ayumi Okizaki, Hideaki Hasuo, Eriko Satomi, Keita Tagami, Kengo Imai, Takashi Kojima, Hironaga Satake, Hiroto Ishiki, Akira Inoue, Takuhiro Yamaguchi

**Affiliations:** ^1^ Department of Palliative Medicine, National Cancer Center Hospital East, Kashiwa, Japan.; ^2^ Innovation Center for Supportive, Palliative and Psychosocial Care, National Cancer Center Hospital, Chuo-ku, Tokyo, Japan.; ^3^ Department of Psychosomatic Medicine, Kansai Medical University, Hirakata, Japan.; ^4^ Department of Palliative Medicine, National Cancer Center Hospital, Tokyo, Japan.; ^5^ Department of Palliative Medicine, Tohoku University Graduate School of Medicine, Sendai, Japan.; ^6^ Seirei Hospice, Seirei Mikatahara General Hospital, Hamamatsu, Japan.; ^7^ Department of Gastroenterology and Gastrointestinal Oncology, National Cancer Center Hospital East, Kashiwa, Japan.; ^8^ Cancer Treatment Center, Kansai Medical University Hospital, Hirakata, Japan.; ^9^ Division of Biostatistics, Tohoku University Graduate School of Medicine, Sendai, Japan.

**Keywords:** appetite, cancer patients, cancer-related fatigue, dexamethasone, inpatient, palliative care

## Abstract

***Objective:*** No standard treatment for cancer-related fatigue (CRF) for inpatients in a palliative care setting exists. The aim of this study was to validate the previous study-derived efficacy of dexamethasone 8 mg for CRF among inpatients in a palliative care setting.

***Methods:*** Inpatients with moderate fatigue (≥4/10) were enrolled in a multicenter phase II trial. Dexamethasone 8 mg p.o. or 6.6 mg i.v. was administered for seven days and 4 mg p.o. or 3.3 mg i.v. for seven consecutive days. The primary endpoint was a threshold average change of Functional Assessment of Chronic Illness Therapy (FACIT)-fatigue subscale score of 3. The secondary endpoints were evaluated with the anorexia-cachexia subscale (ACS), and the Edmonton symptom assessment scale-revised Japanese version.

***Results:*** A total of 32 patients were enrolled. On day 8, the mean change of FACIT-fatigue subscale from day 1 was 5.2 (95% confidence interval 0.8–10.0), in which the lower bound was above 0 but not above the prespecified threshold value of 3.0 (*p* = 0.72). Edmonton symptom assessment system (ESAS)-fatigue was significantly improved by day 3 (*p* = 0.02), but not on day 8 or day 15. ACS, physical well-being, and ESAS-lack of appetite significantly improved by day 8 and day 15. Adverse events were tolerable.

***Conclusion:*** This study showed that dexamethasone 8 mg failed to achieve the preset efficacy for CRF among inpatients in a palliative care setting. However, this treatment improved fatigue and would be an option for CRF.

jRCT (jRCTs031180068).

## Introduction

Cancer-related fatigue (CRF) is observed in 50% to 70% of advanced cancer patients^1^ and is the most distressing symptom of terminally ill cancer patients.^2^ CRF is a multidimensional symptom^3^ and refractory to pharmacologic treatment.^4^ A double-blinded placebo-controlled randomized controlled trial (RCT) using a validated questionnaire among outpatients showed that dexamethasone 8 mg improved CRF.^5^ Another double-blinded placebo-controlled RCT reported that methyl prednisolone 32 mg improved CRF as a secondary endpoint among outpatients.^6^ Among inpatients with CRF and undergoing only palliative care, an observation study showed that a mean betamethasone concentration of 2.4 mg improved CRF using the numeric rating scale (NRS).^7^ In this observation study, palliative care physicians decided the dose of corticosteroids at each physician's discretion. Although more dosage of dexamethasone might show more efficacy even in inpatients with CRF undergoing palliative care, there are no standard treatments for inpatients with CRF undergoing palliative care.

The aim of the phase II clinical trial is to reproduce the same efficacy of dexamethasone 8 mg for CRF among inpatients with CRF who were undergoing palliative care using a validated questionnaire.

## Methods

A multicenter phase II study was conducted to validate the efficacy of dexamethasone 8 mg for CRF in inpatients in a palliative care setting. This study was conducted in accordance with the ethical standards of the Declaration of Helsinki, the Ethical Guidelines for Medical and Health Research Involving Human Subjects, and the Clinical Trials Act by an Ordinance of the Ministry of Health, Labor and Welfare in Japan. This study was approved by the National Cancer Center Hospital East (NCCE) Certified Review Board (CRB3180009) and registered in jRCT.

### Clinical Palliative Care Program

The NCCE is one of five institutes in this study. NCCE is a high-volume cancer center, providing the best in cancer treatment and palliative care. NCCE has four palliative care programs: (1) outpatient clinics for cancer patients at any cancer stage, (2) palliative care consultation for cancer patients on the oncology floor, (3) an inpatient palliative care unit, and (4) consultation service for social or economic issues. Patients and caregivers are referred to these palliative care programs by oncologists or by their own request. NCCE has 8 palliative care physicians, 3 psycho-oncologists, and 52 certificated nurses involved in palliative care.

Kansai Medical University Hospital is an advanced cancer center hospital located in Osaka, a big city in western Japan. The palliative care center offers three main palliative care programs: (1) the outpatients clinics for cancer patients with any cancer stage, (2) palliative care consultation for cancer patients in the oncology floor, and (3) palliative care education for graduate students and community health care providers.

The National Cancer Center Hospital (NCCH) is a tertiary cancer center providing the best cancer treatments and palliative care. NCCH provides three palliative care programs: (1) the outpatients clinics for cancer patients with any cancer stage, (2) palliative care consultation for cancer patients in oncology ward, and (3) consultation about any social or economic issues.

All programs were provided for five days a week. Patients and caregivers were referred to these palliative care programs by their own will or by oncologists. NCCH have 4 palliative care physicians, 4 psycho-oncologists, and 49 certificated nurses involved in palliative care.

Tohoku University Hospital (TUH) that belongs to Tohoku University Graduate School of Medicine has a high-volume palliative care division. TUH set three specialized palliative care services for any patients with any diseases in hospital: (1) outpatients clinics, (2) palliative care consultation for any inpatients, and (3) palliative care unit for cancer and HIV infection patients, and provided specialized palliative care outreach programs to the community and rural areas. Hospital-based outpatients clinic and consultation service were provided for five days a week and palliative care unit for seven days a week, and outreach program is delivered daily in any areas by several health care providers. Patients and caregivers were referred to those specialized palliative care programs by their own will or any health care providers. TUH has 11 palliative care physicians, 2 psycho-oncologists, and 8 certificated nurses involved in our palliative care services.

The Seirei Mikatahara General Hospital is a 934-bed designated cancer hospital and providing cancer treatments and palliative care. It provides three palliative care programs: (1) the outpatients clinics for cancer patients, (2) palliative care consultation for inpatients, and (3) a 27-bed palliative care unit. All programs were provided for five days a week. Patients and caregivers were referred to these palliative care programs by their own will. It has eight palliative care physicians and nine certificated nurses involved in palliative care.

### Subjects

The selection criteria for patients consisted of the following: (1) a cancer diagnosis, (2) 20 years and older, (3) a fatigue intensity score of four or more on a 0 to 10 NRS, (4) a life expectancy >30 days estimated by palliative care physicians, (5) inpatients undergoing only palliative care, (6) no anticancer treatment plan, (7) a performance status that ranged from 0 to 3, and (8) had provided written informed consent. The patients who had (1) received systemic corticosteroid therapies within one week, (2) received surgery within four weeks, (3) received radical irradiation therapy within two weeks, (4) received antitumor treatment within two weeks, and (5) diabetes mellitus, an active infection, or cognitive disorder were ineligible for this study.

### Treatment methods

A physician decided on oral or intravenous dexamethasone treatment. The oral treatment was dexamethasone 8 mg once a day in the morning for days 1 to 7 and 4 mg once a day in the morning for days 8 to 14. The intravenous treatment was dexamethasone 6.6 mg once a day in the morning for days 1 to 7 and 3.3 mg once a day in the morning for days 8 to 14. After starting the treatment, a change of administration route was not permitted.

### Measurements

Patient characteristics were collected from an electronic chart. Each symptom as well as quality of life (QOL) was collected using self-administered questionnaires that included the Functional Assessment of Chronic Illness Therapy (FACIT)-fatigue subscale and Functional Assessment of anorexia/cachexia therapy (FAACT) on days 1, 8, and 15, and the Edmonton symptom assessment system-revised Japanese version (ESASr-J) and fatigue NRS every day, and personalized symptom goal of fatigue (PSG-fatigue) on day 1, and the achievement of PSG-fatigue and patient's satisfaction toward the protocol treatment on days 3, 8, and 15. Adverse events were evaluated using the Common Terminology Criteria for Adverse Events version 4.0 and Confusion Assessment Method (CAM).

The FACIT-fatigue subscale was developed by FACIT.org and is widely used for evaluation of CRF^8,9^ and was used in a previous RCT.^5^ It consists of 13 items using a 0 to 4 Likert scale (0, not at all; 4, very much). FAACT was also developed by FACIT.org and is widely used for the evaluation of anorexia and cachexia.^10^ It consists of 27 general QOL questions (functional assessment of cancer therapy-general), which are divided into 4 domains (physical well-being [PWB], social well-being [SWB], emotional well-being [EWB], and functional well-being [FWB]), and 12 items for the anorexia-cachexia subscale (ACS). These items were evaluated using a 0 to 4 Likert scale (0, not at all; 4, very much).

ESASr-J was used to assess 9 symptoms using a 0 to 10 NRS (0: none–10: worst): 6 physical symptoms (pain, fatigue, nausea, drowsiness, dyspnea, and anorexia), 2 psychological symptoms (depression and anxiety), and well-being.^11^ The time frame of ESASr-J was specified as “now.” The fatigue NRS evaluated the average severity of fatigue during the previous 24 hours using 0 to 10 NRS (0: none–10: worst).^7^

PSG-fatigue was assessed by asking the patients, “at what level would you feel comfortable with fatigue?” to identify the maximal symptom intensity they would consider comfortable on a 0 to 10 NRS.^12,13^ The achievement of PSG-fatigue was defined as fatigue intensity from ESASr-J lower than the PSG-fatigue score.^12,13^

Patient's satisfaction for the protocol treatment was assessed using a 6-point Likert scale ranging from “strongly disagree” to “strongly agree,” and calculated using the proportion of subjects rated “slightly agree,” “agree,” and “strongly agree” among subjects who completed the questionnaires. CAM is a widely used instrument to identify delirium.^14^ Laboratory data within one week from enrollment were obtained from the electronic chart.

### Statistical methods

Continuous data were summarized using median (range), mean (±standard deviation), and mean (95% confidence interval [CI]). General linear models that included a time point (day 0, day 1, and day 8) as explanatory variables were used to summarize the longitudinal change of the FACIT-fatigue subscale scores. An unstructured covariance matrix was assumed, and robust standard error was calculated for parameter estimates. The point estimate and 95% CI were calculated for the mean scores at each time point and the difference at day 8 from day 1. When the lower limit of the CI exceeded the threshold of three points, protocol treatment was used. For the purpose to validating the previous results of Yennurajalingam et al.^5^ in the present population, the required sample size was calculated to be 32 patients for the study to have a power of 90%, assuming that the expected and threshold average change of FACIT-fatigue subscale score was 8 and 3, respectively; a standard deviation of 8; a one-sided significance level of 0.025; and 10% of cases were ineligible. Point estimates and CI s for the average change of other measurements at each day were calculated. Kaplan–Meier methods were used to show the median period of maximum effect of dexamethasone in ESAS-fatigue and ESAS-lack of appetite.

## Results

**FIG. 1.**
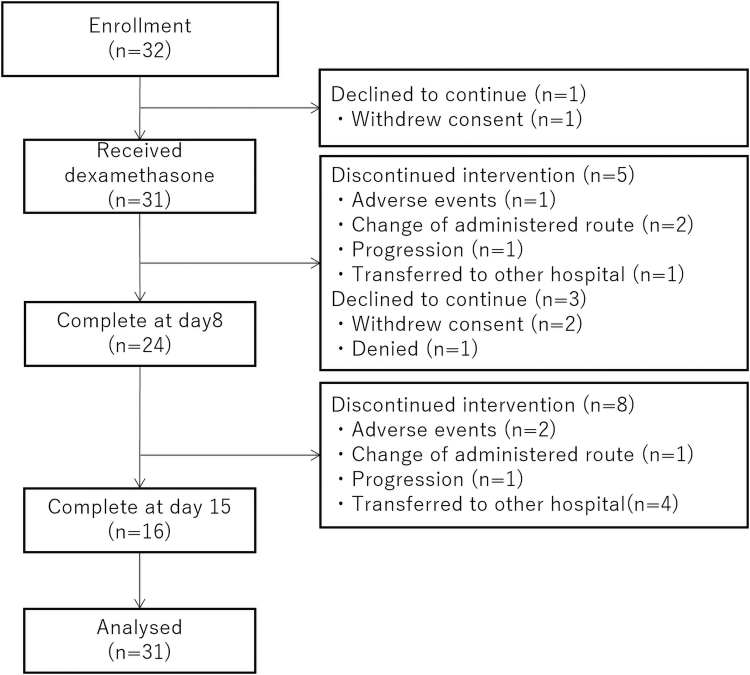
CONSORT diagram.

A total of 32 patients with cancer and an average fatigue intensity of ≥4 were enrolled at five institutes between October 2017 and September 2019. The reasons for excluding patients are shown in Figure 1. A total of 14 patients did not complete the study period: 1 patient withdrew consent before any study procedures were done, 2 withdrew consent during the study period, 1 declined to continue the treatment, 3 discontinued due to adverse events, 3 changed the drug administration route, and 2 discontinued due to disease progression. The patients' backgrounds, symptoms, and laboratory data at day 1 are given in Table 1.

**Table 1.** Patients' Backgrounds

Variables	*N* (%)
Age, median (range)	65.5 (39–83)
Gender	
Male	18 (56.3)
Female	14 (43.7)
Performance status	
0–1	0 (0)
2	6 (18.8)
3	25 (81.2)
Primary tumor site	
Colorectal	9 (29)
Lung	5 (16.1)
Esophagus	5 (16.1)
Pancreas	3 (9.7)
Liver and bile duct	2 (6.5)
Ovary and uterus	2 (6.5)
Breast	1 (3.2)
Stomach	1 (3.2)
Bone and soft tissue	1 (3.2)
Other	2 (6.5)
Distant metastasis	
Lymph node	16 (51.6)
Lung	12 (38.7)
Liver	9 (29.0)
Bone	9 (29.0)
Brain	1 (3.2)
Symptom measures	
FACIT-fatigue subscale	15.5 (11.5)
Fatigue NRS	6.5 (2.3)
FACT-G	50.2 (12.5)
PWB	9.6 (5.0)
SWB	18.1 (5.6)
EWB	12.6 (5.0)
FWB	9.9 (5.0)
ACS	20.8 (8.7)
FAACT total	71 (16.2)
FAACT TOI	40.3 (14.4)
ESAS pain	4.1 (3.1)
ESAS tiredness	5.7 (3.1)
ESAS drowsiness	5.0 (3.1)
ESAS nausea	2.4 (3.1)
ESAS lack of appetite	5.8 (3.7)
ESAS shortness of breath	3.7 (3.7)
ESAS depression	3.9 (3.1)
ESAS anxiety	4.6 (3.1)
ESAS well-being	6.2 (2.5)
ESAS physical distress score	26.7 (15.0)
ESAS psychological distress score	8.5 (5.6)
ESAS symptom distress score	41.4 (21.2)
Laboratory data	
Albumin (g/dL)	2.6 (0.7)
White blood cell ( × 100/μL)	110.3 (74.0)
Hemoglobin (g/dL)	10.3 (1.5)
Platelet ( × 10,000/μL)	33.3 (15.5)
C-reactive protein (mg/dL)	7.6 (5.5)
Aspartate aminotransferase (IU/L)	35 (20)
Alanine aminotransferase (IU/L)	19 (12)
Total bilirubin (mg/dL)	0.58 (0.32)
Creatinin (mg/dL)	0.82 (0.34)

Data are in *n* (%) or mean (SD) unless reported otherwise.

ACS, anorexia-cachexia subscale; ESAS, Edmonton symptom assessment system; EWB, emotional well-being; FAACT, functional assessment of anorexia-cachexia therapy; FACIT, functional assessment of chronic illness therapy; FACT-G, functional assessment of cancer therapy-general; FWB, functional well-being; NRS, numeric rating scale; PWB, physical well-being; SWB, social well-being; TOI, trial outcome index.

### Treatment effect on fatigue

At day 8, the mean FACIT-fatigue subscale score significantly improved [mean change 5.2 (95% CI 0.8–10.0), *p* = 0.02) (Table 2). However, this study failed to achieve the preset efficacy because the lower threshold of average change of FACIT-fatigue subscale was lower than 3.0 (*p* = 0.72). Both fatigue NRS and ESAS-fatigue were significantly improved at day 3 [−1.11 (−2.0 to −0.2), *p* = 0.01 and −1.3 (−2.3 to −0.2), *p* = 0.02, respectively], but these efficacies were not shown at day 8 [−0.76 (−1.8 to 0.3), *p* = 0.16 and −0.5 (−1.7 to 0.6), *p* = 0.38, respectively] and at day 15 [−0.63 (−1.8 to 0.5), *p* = 0.28 and −0.2 (−1.5 to 1.0), *p* = 0.71, respectively]. The achievement of PSG-fatigue was 17.9%, 25.9%, and 18.8% at days 3, 8, and 15, respectively. Overall satisfaction with treatment was 73.1%, 66.7%, and 75.0% at days 3, 8, and 15, respectively. *Ad hoc* analysis showed that the median period of maximum effect for fatigue was day 5 (95% CI days 4–9).

**Table 2.** Changes in Symptoms at Days 3, 8, and 15

	Day 8 change from baseline		Day 8 compared with the preset efficacy	Day 15 change from baseline		Day 3 change from baseline	
	Mean (95% CI)	*p*	*p*	Mean (95% CI)	*p*	Mean (95% CI)	*p*
FACIT-fatigue subscale	5.2 (0.8 to 10.0)	0.02^*^	0.72	3.9 (−1.2 to 9.1)	0.13		
Fatigue NRS	−0.8 (−1.8 to 0.3)	0.16		−0.6 (−1.8 to 0.5)	0.28	−1.1 (−2.0 to −0.2)	0.01^*^
FACT-G	3.2 (−1.8 to 8.2)	0.20		4.6 (−1.8 to 11)	0.16		
PWB	3.3 (1.0 to 5.5)	<0.01^*^		2.9 (0.7 to 5.1)	<0.01^*^		
SWB	0.5 (−0.8 to 1.9)	0.43		0.05 (−1.9 to 1.9)	0.96		
EWB	−0.5 (−2.5 to 1.5)	0.61		0.6 (−2.2 to 3.4)	0.69		
FWB	0.4 (−2.0 to 2.9)	0.72		1.1 (−1.6 to 3.7)	0.42		
ACS	5.6 (2.5 to 8.8)	<0.01^*^		5.2 (0.6 to 9.7)	0.03^*^		
FAACT total	8.8 (2.2 to 15.5)	0.01^*^		9.7 (0.1 to 19.4)	0.05^*^		
FAACT TOI	9.3 (3.5 to 15.2)	<0.01^*^		9.1 (1.5 to 16.7)	<0.01^*^		
ESAS pain	−1.3 (−2.3 to −0.3)	0.02^*^		−0.7 (−1.8 to 0.4)	0.23	−1.2 (−2.1 to −0.3)	0.01^*^
ESAS tiredness	−0.5 (−1.7 to 0.6)	0.38		−0.2 (−1.5 to 1.0)	0.71	−1.3 (−2.3 to −0.2)	0.02^*^
ESAS drowsiness	−1.3 (−2.4 to −0.2)	0.03^*^		−0.8 (−2.1 to 0.6)	0.28	−0.8 (−1.8 to 0.2)	0.13
ESAS nausea	−0.6 (−1.4 to 2.0)	0.14		−0.6 (−1.6 to 0.5)	0.31	−0.6 (−1.7 to 0.5)	0.27
ESAS lack of appetite	−1.6 (−2.9 to −0.2)	0.02^*^		−1.7 (−3.3 to −0.1)	0.04^*^	−1.9 (−3.1 to −0.7)	<0.01^*^
ESAS shortness of breath	0.2 (−1.0 to 1.4)	0.76		0.2 (−1.2 to 1.7)	0.78	−0.6 (−1.6 to 0.5)	0.28
ESAS depression	0.5 (−0.9 to 2.0)	0.45		0.008 (−1.5 to 1.5)	0.99	−0.7 (−1.6 to 0.1)	0.10
ESAS anxiety	−0.004 (−1.4 to 1.4)	1.00		−0.5 (−2.1 to 1.1)	0.53	−1.3 (−2.3 to −0.2)	0.02^*^
ESAS well-being	−1.1 (−2.4 to 0.1)	0.08		−0.9 (−2.3 to 0.4)	0.18	−1.8 (−2.6 to −1.0)	<0.01^*^
ESAS physical distress score	−5.1 (−10.2 to −0.1)	0.05^*^		−3.7 (−9.6 to 2.2)	0.22	−6.3 (−10.8 to −1.8)	<0.01^*^
ESAS psychological distress score	0.5 (−2.1 to 3.2)	0.69		−0.5 (−3.5 to 2.5)	0.74	−2 (−3.9 to −0.1)	0.04^*^
ESAS symptom distress score	−5.7 (−14.2 to 2.8)	0.19		−5.1 (−14.6 to 4.4)	0.29	−10.1 (−16.3 to −3.9)	<0.01^*^

^*^ ; < 0.05 compared to baseline.

CI, confidence interval.

### Other symptoms

ESAS-lack of appetite significantly improved at day 3 [−1.9 (−3.1 to −0.7), *p* < 0.01] and this improvement continued to day 8 [−1.6 (−2.9 to −0.2), *p* = 0.02] and day 15 [−1.7 (−3.3 to −0.1), *p* = 0.04]. *Ad hoc* analysis showed that the median period of maximum effect for lack of appetite was day 4 (95% CI days 3–6). ESAS-pain improved at day 3 [−1.2 (−2.1 to −0.3), *p* = 0.01] and day 8 [−1.3 (−2.3 to −0.3), *p* = 0.02]. ESAS-anxiety and ESAS-well-being improved at day 3 [−1.3 (−2.3 to −0.2), *p* = 0.02, and −1.8 (−2.6 to −1.0), *p* < 0.01, respectively]. *Ad hoc* analysis showed that the median period of maximum effect for lack of appetite was day 4 (95% CI days 3–6).

### Quality of life

ACS had significantly improved at day 8 [5.6 (2.5–8.8), *p* < 0.001] and day 15 [5.2 (0.6–9.7), *p* = 0.03]. PWB had also significantly improved at day 8 [3.3 (1.0–5.5), *p* < 0.01] and day 15 [2.9 (0.7–5.1), *p* < 0.01]. However, SWB, EWB, and FWB did not improve.

### Adverse events

A total of 29 adverse events occurred (Table 3). Among them, seven were severe adverse events with a grade of 3 or 4 (fatigue *n* = 3, delirium *n* = 1, hyperglycemia *n* = 1, anemia *n* = 1, and insomnia *n* = 1). The patients with fatigue and delirium died because of disease progression. None of the adverse events were suspected to have been caused by the study medication. CAM identified one patient until day 8 and four patients until day 15 with delirium.

**Table 3.** Adverse Events in Dexamethasone Treatment

	All grades	Grade 3 or 4
	*N* (%)	*N* (%)
Delirium	2 (6.5)	1 (3.2)
Gastric hemorrhage	1 (3.2)	0 (0)
Cough	1 (3.2)	0 (0)
Generalized muscle weakness	1 (3.2)	0 (0)
Somnolence	6 (19.4)	0 (0)
Hyperglycemia	2 (6.5)	1 (3.2)
Hyperhidrosis	1 (3.2)	0 (0)
Fatigue	3 (9.7)	3 (9.7)
Anemia	1 (3.2)	1 (3.2)
Insomnia	10 (32.3)	1 (3.2)
Edema limbs	1 (3.2)	0 (0)
Total	29	7

## Discussion

This study showed that dexamethasone 8 mg for CRF in inpatients without an antitumor treatment plan failed to achieve preset efficacy, which we defined as the placebo effect using the criteria of the previous RCT.^5^ Dexamethasone 8 mg improved FACIT-fatigue subscale, lack of appetite, ACS, and PWB. In addition, adverse events were tolerable.

The most important finding was that the efficacy of dexamethasone for CRF was decreased among this study population compared with the previous RCT.^5^ The efficacy of dexamethasone may vary depending on the patients' general condition. Among this study population, three-quarters of participants were performance status (PS) 3 and mean C-reactive protein (CRP) was high (7 mg/dL). In contrast, the previous RCT included no information about PS, FWB, or laboratory data.^5^ In addition, the general condition in the previous RCT may be better than those in this study population because patients who received any treatment were eligible and the FACIT-fatigue subscale at baseline was higher than that in this study. In contrast, Miura et al. reported similar FACIT-fatigue subscale and CRP level scores to the inpatients in our palliative care unit (PCU)^15^ (data not shown). Matsuo et al. reported that systemic corticosteroids (mean betamethasone: 2.4 mg/day) improved fatigue NRS score by 1.9,^7^ which was greater than those in this study. This study excluded patients with PS 4, which was associated with nonrespondence to systemic corticosteroids.^7^ However, the change of fatigue NRS in this study was lower. The mentioned differences may be due to the data collection method as this study used self-administered questionnaires, which the other studies did not use. The differences may also be explained by inflammatory status as the levels of CRP and albumin in that study^7^ were 5 mg/dL and 2.8 g/dL, respectively, which were lower and higher than those in this study. Albumin level is considered as an inflammatory marker because inflammation stimulates hepatocytes to increase acute phase protein synthesis and to decrease albumin synthesis.^16^ Therefore, this study population may be under severe and continuous inflammatory conditions, compared with that study.^7^ Newton et al. reported that glucocorticoid and cytokine crosstalk regulated repression or resistance to the effect of corticosteroid therapy.^17^ Under inflammation-induced oxidative stress, the decrease of the activity and expression of histone deacetylase-2, which was needed to activate inflammatory genes, becomes resistant to the anti-inflammatory actions of glucocorticoids.^17–20^

In this study population, the short-term administration of high-dose dexamethasone improved ACS scores as well as lack of appetite and PWB, and did not affect patients' FWB. The present results are similar to previous RCTs using dexamethasone 8 mg^5^ or methylprednisolone (mPSL) 32 mg^6^; however, these studies did not set appetite as a primary endpoint. This study found that the median period of maximum effect for appetite was day 4, which was shorter than that for fatigue (day 5). Thus, appetite may be more responsive to corticosteroid therapy than fatigue. However, even if high-dose dexamethasone stimulates the patient's appetite, dexamethasone should be gradually decreased, as muscle weakness and atrophy are well-known side effects of systemic corticoid treatments.^21^ Considering the long-term effects, improving muscle strength and muscle mass may require a combination of appetite stimulants, such as short-term administration of high-dose dexamethasone, and anticachexic agents, such as anamoreline, in future trials. The effect of dexamethasone and anamoreline as appetite stimulants could enable advanced cancer patients to keep living in their own home with home-based palliative care.

This study has several limitations. The first is limitations regarding generalization. This study had strict criteria for inclusion and exclusion. Therefore, it took 2.2 years to enroll 32 participants. The second is sampling methods. This study had convenient sampling and not continuous sampling; therefore, the reasons why patients were ineligible were not investigated. The third is a high attrition rate. This study showed 43.8% of subjects did not complete a two-week study protocol. Whereas the inpatients undergoing only palliative care might rapidly worsen their general conditions, these might affect results. The fourth is the lack of a placebo arm. Present results were affected by placebo effects, even though the primary outcome was taking into account the placebo effect of the prior dexamethasone for CRF outpatients' study. The fifth is that timing of primary outcome was set on day 8, although previous RCTs set timing of primary outcome on day 15. Subjects enrolled into this study were inpatients, which might be a worse general condition than those of previous RCTs. This population was quite frail and easy to evoke delirium. Palliative care physicians paid attention to the risk of delirium. Therefore, we considered short-term outcome (day 8 outcome) as appropriate and acceptable, and we reduced the dexamethasone dosage from day 8 to day 14. The sixth is that physicians decided treatment routes for subjects. The subjects who received dexamethasone 6.6 mg i.v. might show worse conditions than those who receiced dexamethasone 8 mg p.o. The seventh is study setting. The previous RCT was performed for outpatients^5^; however, this study was for inpatients. However, mean changes of fatigue NRS showed similar levels to the previous RCT^5^ and previous observation studies^7,13^; therefore, we were able to create this study design using the RCT's criteria. This study failed to validate the RCT's results; however, dexamethasone 8 mg p.o. or 6.6 mg i.v. improved the fatigue score among inpatients undergoing palliative care. This treatment may be an option for inpatients with moderate CRF.

## Conclusions

This study failed to achieve the preset efficacy. However, dexamethasone 8 mg improved fatigue, appetite, ACS, and PWB, and was tolerable among inpatients undergoing only palliative care. This treatment may be an option for inpatients with moderate CRF.

## Abbreviations Used

ACS: anorexia-cachexia subscale
CAM: Confusion Assessment Method
CI: confidence interval
CRF: cancer-related fatigue
CRP: C-reactive protein
ESAS: Edmonton symptom assessment system
ESASr-J: Edmonton symptom assessment system-revised Japanese version
EWB: emotional well-being
FAACT: Functional Assessment of Anorexia-Cachexia Therapy
FACIT: Functional Assessment of Chronic Illness Therapy
FACT-G: Functional Assessment of Cancer Therapy-General
FWB: functional well-being
NCCE: National Cancer Center Hospital East
NCCH: National Cancer Center Hospital
NRS: numeric rating scale
PS: performance status
PSG: personalized symptom goal
PWB: physical well-being
QOL: quality of life
RCT: randomized controlled trial
SWB: social well-being
TOI: trial outcome index
TUH: Tohoku University Hospital
